# A Goodwin Model Modification and Its Interactions in Complex Networks

**DOI:** 10.3390/e25060894

**Published:** 2023-06-02

**Authors:** Francisco Yáñez Rodríguez, Alberto P. Muñuzuri

**Affiliations:** 1 Group of NonLinear Physics, University of Santiago de Compostela, 15706 Santiago de Compostela, Spain; francisco.yanez@rai.usc.es; 2 Galician Center for Mathematical Research and Technology (CITMAga), 15782 Santiago de Compostela, Spain

**Keywords:** econophysics, nonlinear interactions, dynamical systems, complex networks

## Abstract

The global economy cannot be understood without the interaction of smaller-scale economies. We addressed this issue by considering a simplified economic model that still preserves the basic features, and analyzed the interaction of a set of such economies and the collective emerging dynamic. The topological structure of the economies’ network appears to correlate with the collective properties observed. In particular, the strength of the coupling between the different networks as well as the specific connectivity of each node happen to play a crucial role in the determination of the final state.

## 1. Introduction

Much attention has been devoted to the synergetic behaviors of sets of coupled dynamical systems and their nonlinear interactions. Examples are widely observed, ranging from biology [1,2,3,4], chemistry [5,6,7,8,9,10], social systems [11,12], and, of course, economy [13,14,15]. In all these cases, the collective phenomena observed are more than just the addition of the individuals, and new collective dynamics emerge.

The structure of the network of connections has shown to be determinant in the collective phenomena observed. With this manuscript, we plan to show the role played by the network topology on the synergetic properties observed in the economic models. For that, we considered a set of simple economic models coupled.

Although there are many models that describe different aspects of an economy, such as the Harrod–Domar model [16,17], the Solow–Swan model [18], and the Philips curve model [19], one of the most known models in economics is the Goodwin model [20], which focuses on predicting salary and employment. This model is based on the very-well-known Lotka–Volterra model, used to predict the relation between prey and predators [21] in population dynamic studies. In the Goodwin model, salary plays the role of the predator whereas employment is the prey, exhibiting periodic behaviors. This model combines many of the properties of some of the previous models [22] and its equations exhibit a relatively simple dynamic so that the outcome of coupling several such economies becomes more apparent.

This simple model has been extended in many different contributions (a summary of these can be found in [23,24,25]) aiming to expand its applicability by considering different scenarios. In these extensions, different dynamics were included and even empirical data seem to be described. With this manuscript, we stuck to the original version of the model and only included a minor modification that prevents the appearance of non-physical divergent solutions. Thus, the long-term divergent behaviors (exponential growths) were erased in order to analyze the global dynamics in a medium-to-long-term temporal scale. This guarantees that all the variables do not diverge and also adds more possible solutions and behaviors to the economies predicted. In this way, the focus of this contribution is on the collective phenomena arising as several economies interact together, exchanging resources in a network. Note that this is particularly relevant nowadays as globalization has forced economies to interact with each other, but we are not yet in a global world with one single macroeconomy and thus the network structure and strength play a significant role.

The paper is organized as follows. The methods section presents the Goodwin model with the modifications introduced and a description of the dynamical behaviors that can be observed. The second part of the methods section presents the different network topologies that we considered. The results and discussions of our study are presented in the next section and the manuscript is ended with some conclusions.

## 2. Materials and Methods

### 2.1. Goodwin Model and Corrections

The original Goodwin model is an attempt to describe the behavior of salary and employment [20,22]. With that purpose, it is necessary to define the following variables:

- $c\left(t\right)$ is the capital invested in the production of a good.
- $q\left(t\right)$ is the quality of the final product. A linear relation with the capital invested is assumed, so we can write $c\left(t\right)=\kappa \cdot q\left(t\right)$.
- $n\left(t\right)$ is the population involved in the production of a good.
- $p\left(t\right)$ is the productivity of employees.
- $w\left(t\right)$ is the salary that a single subject receives when working in the production of a good, called unit wage in the following.
- $l\left(t\right)\equiv q\left(t\right)/p\left(t\right)$ is the ratio of quality and productivity. It relates both variables and provides information about the effectiveness of the jobs from the point of view of the producers, which we will name employment level.
- $u\left(t\right)\equiv w\left(t\right)/p\left(t\right)$ is the ratio between salary and productivity. We will refer to it as the salary directly.
- $v\left(t\right)\equiv l\left(t\right)/n\left(t\right)$ is the ratio between the level of employment and the number of employees, or the employment rate.

The set of Equation (1) describes the original model first introduced by Richard Goodwin [20]:

(1) $$\begin{array}{c} \frac{dp}{dt}=\alpha \;p\\ \frac{dw}{dt}=w\left(\rho \;v-\gamma \right)\\ \frac{dn}{dt}=\delta \;n\\ \frac{dc}{dt}=q\left(1-u\right) \end{array}\begin{array}{c} \frac{dq}{dt}=q\left(\frac{1-u}{\kappa }\right)\\ \frac{dl}{dt}=l\left(\frac{1-u}{\kappa }-\alpha \right)\\ \frac{dv}{dt}=v\left(\frac{1-u}{\kappa }-\alpha -\delta \right)\\ \frac{du}{dt}=u\left(\rho \;v-\gamma -\alpha \right) \end{array}$$

As we can see in Equation (1), Richard Goodwin’s original model considers that population and productivity exponentially grow with time and thus most of the model variables also tend to infinite for sufficiently long times.

In this set of equations, the variables are those described above, and the parameters introduced are ρ,γ,κ,α, and δ. Note that ρ and γ already introduce some limitations to the salary values as they tend to prevent the exponential growth (this is not fully achieved as the productivity might exponentially grow and thus force the whole system to diverge). The parameters δ and α will determine how fast productivity and population grow, as they control the exponential growth in the equations. On the other hand, ρ and γ are parameters that characterize the time evolution of the unitary salary w and thus the salary u. Finally, ρ and γ quantify the dependence of the employment on the evolution of the salary, playing a similar role to that in the previously mentioned Philips curve [19]. Finally, κ is the relation between capital and quality mentioned above. All these parameters are defined as positive. Some examples of the dynamical behaviors observed are presented in the Supplementary Materials.

To avoid the exponential growths observed in the original Goodwin model, we introduced a second-order correction term in the sense of the logistic equation, i.e., when the values of the variables start to significantly grow, the added terms prevent divergencies by assuming a limited number of resources. The new set of equations, which will be used in this manuscript, is given by

(2) $$\begin{array}{c} \frac{dn}{dt}=0\\ \frac{dp}{dt}=\alpha \;p\left(1-\frac{p}{\sigma }\right)\\ \frac{dw}{dt}=\left(w-\beta_{u}\right)\left(\rho \;v-\gamma \right)\\ \frac{dc}{dt}=\left(q-\beta_{v}\right)\left(1-\lambda \;u\right) \end{array}$$

where the parameters α and σ control the logistic growth, σ determines the value of the productivity compatible with the considered economy, and α is the productivity growth rate. We also introduced the parameters $\beta_{u}$ and $\beta_{v},$ which are related to some physical constraints in the salary and employment variables that we will discuss in more details below. λ is another parameter introduced to control the evolution of the capital and avoid divergencies. Finally, ρ and γ take the same role as in the original model. In addition, as in the previous model, all the parameters introduced by us were constrained to positive values. Note that this approach to limiting the exponential growth of the system is the simplest that can be considered, and it just limits the resources at our disposal.

Supposing that the relation between capital and quality is linear ($c\left(t\right)=\kappa \cdot q\left(t\right)$), the other variables in the model are then given by the following Equation (3):

(3) $$\begin{array}{c} \frac{dq}{dt}=\left(q-\beta_{v}\right)\frac{1-\lambda u}{\kappa }\\ \frac{dl}{dt}=\left(l-\frac{\beta_{v}}{p}\right)\frac{1-\lambda u}{\kappa }-\alpha l\left(1-\frac{p}{\sigma }\right)\\ \frac{dv}{dt}=\left(v-\frac{\beta_{v}}{np}\right)\frac{1-\lambda u}{\kappa }-\alpha v\left(1-\frac{p}{\sigma }\right)\\ \frac{du}{dt}=\left(u-\frac{\beta_{u}}{p}\right)\left(\rho v-\gamma \right)-\alpha u\left(1-\frac{p}{\sigma }\right) \end{array}$$

**Figure 1:**
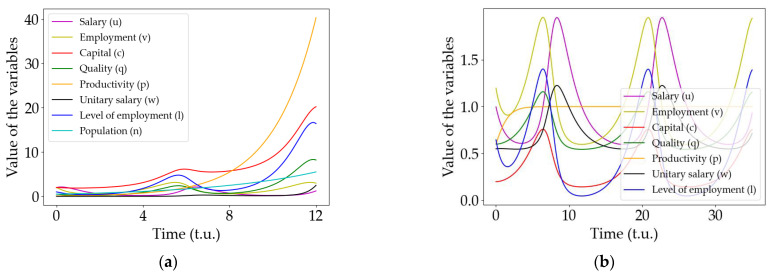
Comparison between the predictions of the original model and our modification (given by Equations (2) and (3)). (**a**) Solution to the equations of the original Goodwin model. Note that the exponential growth of the productivity (the orange curve), makes the oscillations amplitude of the other variables diverge. (**b**) Solution of the modified model. Note that the oscillations remain stable in time due to the logistic behavior of the productivity variable (in color orange).

Figure 1 presents numerical simulations of the original Goodwin model (Figure 1a) and the modified model (Figure 1b), both obtained using an explicit 4th-order Runge–Kutta integration scheme [26]. It is possible to observe that the oscillatory behavior remains stable even for long periods of time whereas the original model steadily diverges. Note that the original Goodwin model in Figure 1a presents an exponential growth of the variables as time evolves (this is clearly seen in the orange curve, employment). On the other hand, once the modification is included, the exponential growth is avoided and the oscillatory behavior can be observed for very long periods of time (notice the orange curve in Figure 1b that saturates as expected from Equation (3)).

Note that the oscillatory behavior observed is the same as that predicted in the original Goodwin model; however, in our case, the productivity variable does not diverge and, consequently, the oscillations remain stable, with time allowing for long-time observations and thus interactions between different economies.

From now on, we will focus on the behaviors of the variables describing wages (u) and employment (v) in a case where the productivity is constant with value p=σ for simplicity, i.e., the productivity for the time considered remains almost constant. Note that the modified set of equations presents a stable point at p=σ; thus, we assume that the productivity has reached the expected stationary value and we consider modifications from this moment. Thus, our set of equations becomes

(4) $$\begin{array}{c} \frac{du}{dt}=f\left(u,v\right)=\left(u-\frac{\beta_{u}}{\sigma }\right)\left(\rho v-\gamma \right)\\ \frac{dv}{dt}=h\left(u,v\right)=\left(v-\frac{\beta_{v}}{n\sigma }\right)\frac{1-\lambda u}{\kappa } \end{array}$$

### 2.2. Linear Stability Analysis and Phase Diagrams

In this section and using linear stability analysis [27], we studied the different behaviors that the set of Equation (4) exhibit. The system presents two fixed points:

(5) $$\left(u_{0}^{1}=\rho /\gamma ,v_{0}^{1}=1/\lambda \right);\left(u_{0}^{2}=\beta_{u}/\sigma ,v_{0}^{2}=\beta_{v}⁄n\sigma \right)$$

In Equation (5), we recover the fixed point $\left(u_{0}^{1},v_{0}^{1}\right)$ that was already present in the original Goodwin model, while $\left(u_{0}^{2},v_{0}^{2}\right)$ depends on the new parameters introduced ($\beta_{u}$ and $\beta_{v}$) and that were not present in the original model. Analyzing the sign of the eigenvalues $e_{i}$ of the Jacobian matrix for our system, we can classify the different behaviors of the system. The Jacobian matrix is given by,

(6) $$\left|J\left(\vec{x}\right)-eI\right|={\left|\begin{array}{cc} \frac{\partial f(u,v)}{\partial u}-e & \frac{\partial f(u,v)}{\partial u}\\ \frac{\partial h(u,v)}{\partial u} & \frac{\partial h(u,v)}{\partial v}-e \end{array}\right|}_{\left(u_{0},v_{0}\right)}$$

Thus, the eigenvalues for each fixed point are given by the expressions

(7) $$\begin{array}{c} \left(u_{0}^{1},v_{0}^{1}\right)\to e_{1,2}^{1}=\pm i\sqrt{\frac{\rho \lambda }{\kappa }\left(\frac{1}{\lambda }-\frac{\beta_{u}}{\sigma }\right)\left(\frac{\gamma }{\rho }-\frac{\beta_{v}}{n\sigma }\right)}\\ \left(u_{0}^{2},v_{0}^{2}\right)\to e_{1}^{2}=\frac{\rho \beta_{v}}{n\sigma }-\gamma ;e_{2}^{2}=\frac{\sigma -\lambda \beta_{u}}{\sigma \kappa } \end{array}$$

**Figure 2:**
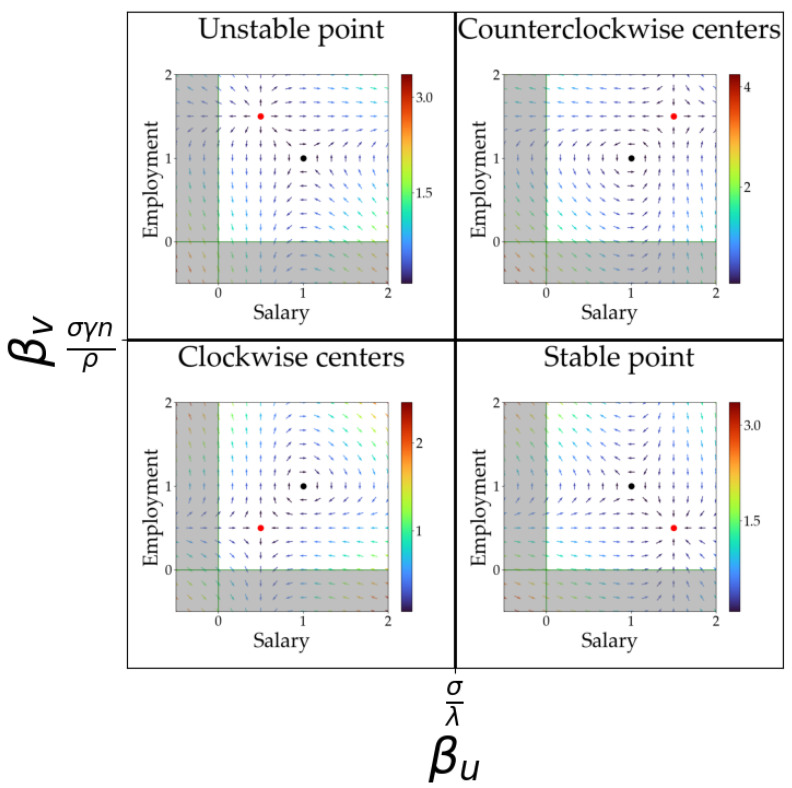
Phase diagram for the modified Goodwin model depending on the parameters ($\beta_{u}$ and $\beta_{v}$). The black spot in each phase diagram marks the position of the first fixed point $\left(u_{0}^{1},v_{0}^{1}\right)$ (always in $\left(1,1\right)$) whereas the red spot marks the second fixed point $\left(u_{0}^{2},v_{0}^{2}\right)$. Shaded regions correspond to negative values for u and/or v and thus do not provide solutions with a physical meaning. All the parameters of the model (including population) are set as equal to 1. The color of the arrows in the diagram reflects the magnitude of the flow vector in the phase diagram (the colder the color, the smaller its magnitude, as indicated in the color bar).

Analyzing the results of Equation (7), we notice that $\beta_{u}$ and $\beta_{v}$ determine the sign of both eigenvalues for each fixed point, resulting in four different behaviors. Note that the sign of the eigenvalues determines the stability of each fixed point and thus the dynamics of the solutions around it. To visualize the different dynamics observed depending on the value of the parameters $\beta_{u}$ and $\beta_{v}$, we present Figure 2. Here, for the different values of $\beta_{u}$ and $\beta_{v}$, we present the flow diagrams for the salary (u) and the employment (v). Note that there are some critical values for the parameters $\beta_{u}=\sigma /\lambda$ and $\beta_{v}=\sigma \gamma n/\rho$ that signal a change in the dynamical behavior and thus a bifurcation point. These values mark the frontier between the different states of our economies.

**Figure 3:**
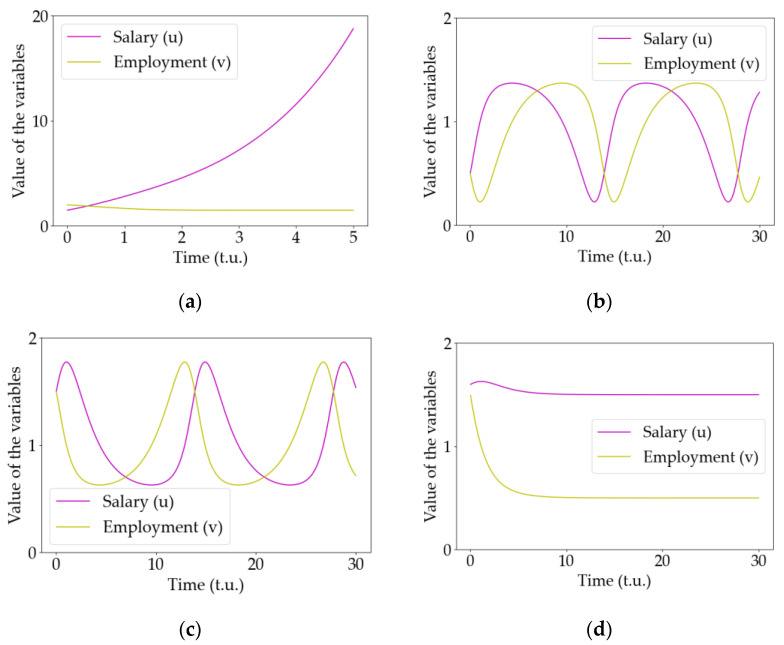
Temporal evolution of the model variables (the salary u and the employment v) for the four different configurations plotted in Figure 1. (**a**) Unstable point with $\beta_{u}=0.5$ and $\beta_{v}=1.5$ (note that the scale in the vertical axis is much larger than those in the others to stress the exponential behavior of this solution). (**b**) Counterclockwise (CCW) periodic center with $\beta_{u}=1.5$ and $\beta_{v}=1.5$. (**c**) Clockwise (CW) center with $\beta_{u}=0.5$ and $\beta_{v}=0.5$, corresponding to the original solution to the Goodwin model. (**d**) Stable point with $\beta_{u}=1.5$ and $\beta_{v}=0.5$. The remaining parameters are set as equal to 1.

Figure 3 shows a summary of all the behaviors observed with the modified Goodwin model, integrating the model equations with some initial conditions, where the parameters of the model are equal to 1 except for the beta parameters $\beta_{u}$ and $\beta_{v}$. In this case, it is trivial to see that the bifurcation is located on $\beta_{u}=1$ and $\beta_{v}=1$.

Figure 3a is the temporal evolution of the u and v variables for the model parameters $\beta_{u}=0.5$ and $\beta_{v}=1.5$. This is an unstable situation and, after some transient, the values of the variables diverge; thus, it represents a non-realistic situation without equivalent within the economic context.

Increasing $\beta_{u}$ above the bifurcation point (at $\beta_{u}=1$) produces a completely different dynamic that is plotted in the right upper corner in Figure 2 ($\beta_{u}=1.5$ and $\beta_{v}=1.5$). A counterclockwise center in the flow diagram that translates into a periodic oscillatory behavior is shown in Figure 3b. This situation corresponds to some cyclic dynamic in the economy considered. In economic terms, these are solutions with the same economic meaning as the equivalent predicted by Goodwin in the original model, but with the variables’ roles swapped, i.e., in Goodwin model, the salary is the predator and employment the prey, thinking in the analogue of a Lotka–Volterra model, whereas, in the situation described by Figure 3b, it is the opposite: salary is the prey and employment is the predator, which is something totally different from the previous ideas held in this type of economic theory [22], but with economic meaning. Another important detail is that these oscillations may pass through negative values if the centers are far from the fixed point. However, in these situations, the variations in salary and employment would be enormous in small fractions of time, so these situations are non-realistic. Note that the model proposed is just an approximation of reality within a certain range of validity. In this case, large values of the salaries or employment produce non-realistic solutions and thus we should include additional higher-order terms in the equations if we were to control it. In summary, near the fixed point, the results describe physically and economically sound situations (within the range of validity of the model) that will be analyzed in the coming sections.

Crossing the bifurcation line below $\beta_{v}=1$, different dynamics are observed. In the bottom left corner of Figure 2 ($\beta_{u}=0.5$ and $\beta_{v}=0.5$.), a clockwise center is observed that represents a periodic behavior of the model variables but with completely different period and geometry, analogous to those oscillations predicted by Goodwin in the original model, as shown in Figure 3c. In addition, notice that, with this configuration, the other unstable fixed point prevents these oscillations from crossing into negative values for the variables.

Finally, the bottom right corner of Figure 2 shows the dynamic for $\beta_{u}=1.5$ and $\beta_{v}=0.5$. Here, the system exhibits a stable fixed point, represented in Figure 3d. The economic interpretation is straightforward: the economy will tend to stability and, once it is reached, it remains there without any further dynamic.

From the observation of the phase diagrams in Figure 2, we notice that some other unstable dynamics can be expected, but we will not focus on them as they correspond to unrealistic non-physical situations.

Although the parameters $\beta_{u}$ and $\beta_{v}$ were introduced for mathematical reasons, their meaning in economic terms can be understood based on the dynamics observed. In the Goodwin model solutions (Figure 2 and Figure 3c), they are directly related to minimum values for the two variables: wages (u) and employment (v). This is related to economies where neither the employment nor the salaries can drop to zero. It does not seem a very strong restriction but, nevertheless, it significantly enriches the range of behaviors observed.

### 2.3. Network Topologies

In the following, we considered N economies that are interconnected following a given network structure. We can describe this system using a complex network [28,29,30] where the nodes are each of the N economies considered and the links connecting two nodes describe the existence of an influence relationship between both.

The structure of the interaction network between the different economies considered is fully encoded in the adjacency matrix [28,29,30], where $a_{ij}=1$ if there exists an interaction between economy i and economy j, and 0 otherwise. Since the interaction between two economies is mutual, the adjacency matrix must be symmetric; thus, $a_{ij}=a_{ji}$. We recovered the activity of each node by summing up its interactions in what is called the connectivity degree, or simply degree, $k_{i}$, for each node.

In this manuscript, we considered different types of networks [28,29,30] corresponding to different patterns of influence among the economies:

- A scale-free network (commonly known as Barabasi–Albert). The BA graph mixes network growth and preferential attachment to generate a power-law connectivity distribution. In this case, a few of the nodes are connected with many economies whereas the majority have a significantly reduced number of connections, and a few of the economies are widely connected whereas most of them only have a few connections, mostly with those dominating economies.
- A Watts–Strogatz network. The WS graph is built from an initial chain with k nearest neighbors from which connections are then rewired randomly with a given probability, creating shortcuts along the network. In this case, economies are just connected with neighboring ones with an additional probability to connect to far-away economies. In this configuration, all the economies are connected with the same degree. Most of the connections are local except for a few long-range connections that still describe a proximity economy with some attempts to become global.
- Mean field network. All nodes interact with an imaginary node that is just the average of all the nodes in the network. The specific relationship between all the economies involved is not clear; nevertheless, they all interact through this imaginary node with the mean value. This is a global situation where economies are so interconnected that, finally, they just feel the average among all of them.

The adjacency matrices for all the cases considered were generated using the graph generators of the Python library networkx (specifically the functions nx.watts_strogatz_graph() and nx.barabasi_albert_graph()). It is important to mention that we simulated random networks using a Watts–Strogatz network with p=1 due to the fact that, in a random network, the connectivity for each node is also random, which is something we do not desire. In addition, in this work, we considered that the Watts–Strogatz network has a value of p equal to 0.05, unless stated otherwise. Examples of the networks used and some details are shown in the Supplementary Materials (Section S2).

### 2.4. Economies Connected via a Network

In this section, we present the modifications that were introduced in the model to account for the influence of the other economies. We considered a set of N economies, each of them described by the modified Goodwin model presented in Equation (4). Now, the evolution of the variables for each economy will depend on the internal dynamic but also on the values of the variables of the economies directly connected. This is represented by the following set of equations:

(8) $$\begin{array}{c} \frac{du_{i}}{dt}=\left(u_{i}-\frac{{\beta_{u}}_{i}}{\sigma_{i}}\right)\left(\rho_{i}v_{i}-\gamma_{i}\right)+g\sum_{j=1}^{N}a_{ij}\left(u_{j}-u_{i}\right)\\ \frac{dv_{i}}{dt}=\left(v_{i}-\frac{{\beta_{v}}_{i}}{n_{i}\sigma_{i}}\right)\frac{1-\lambda_{i}u_{i}}{\kappa_{i}}+g\sum_{j=1}^{N}a_{ij}\left(v_{j}-v_{i}\right) \end{array}$$

where i=1…N denotes each of the N economies considered.

The first term of Equation (8) is given by the internal dynamic of each economy that is described in Equation (4). However, the second term carries the information of the network. $a_{ij}$ is an element of the adjacency matrix as described in the previous section that equals 1 when the economies i and j are connected and equals 0 when there is no such connection. The specific shape of the network term is directly derived from Fick’s law, which describes the tendency to displace the excess from one node to a connected deficitary one. Within the economic context of the present model, this means that economies with higher wages or employment rates will influence their connected nodes by raising their equivalent variables. This, as a first approximation, seems reasonable as the well-being of economies tends to positively influence those economies connected (and vice versa). If the employment rates are high in one economy, this will most likely result in an increase in the employability in the connected economies, as well as the salaries. The simplest way to include this in the equations is through the term $u_{j}-u_{i}$, meaning that if the variable $u_{j}$ in the j-node has a higher value, the variable $u_{i}$ will increase (as the derivative $\frac{du_{i}}{dt}>0$). On the other hand, considering the j-node, the situation is the opposite and $\frac{du_{j}}{dt}<0$; thus, the variable $u_{j}$ will be reduced. This is also reasonable as large salaries in an economy surrounded by economies with lower salaries will tend to lower to compensate for the possibility of moving the economy to those neighboring economies. At the same time, the salaries in those neighboring economies will tend to rise until some equilibrium is reached.

The parameter g controls the weight of the neighboring economies (the smaller this term is, the less relevant the network interaction will be). The limiting case with g=0 corresponds to a set of independent economies that do not interfere with each other. Note that those nodes connected with a larger number of nodes will experience a stronger network influence as the number of terms from the summation that are non-null will be larger. This is also reasonable as those economies that interact with a large number of other economies (nodes) will also experience a larger influence from them.

The mathematical description of the network presented is the simplest that can be considered. More complicated interdependencies can be introduced even considering asymmetric relationships between the economies involved [31,32] but this configuration is the simplest that still produces some meaningful results. Note that each node receives a different contribution from the network term as its connectivity might be different.

When a mean field interaction between the economies is considered, the set of equations presented in Equation (8) becomes

(9) $$\begin{array}{c} u_{i}^{\prime }=\left(u_{i}-\frac{{\beta_{u}}_{i}}{\sigma_{i}}\right)\left(\rho_{i}v_{i}-\gamma_{i}\right)+g\left(\langle u\rangle -u_{i}\right)\\ v_{i}^{\prime }=\left(v_{i}-\frac{{\beta_{v}}_{i}}{n_{i}\sigma_{i}}\right)\frac{1-\lambda_{i}u_{i}}{\kappa_{i}}+g\left(\langle v\rangle -v_{i}\right) \end{array}$$

where $\langle u\rangle$ and $\langle v\rangle$ are the average value over all the considered economies of the u and v variables, respectively. Note that these equations describe the interaction of each node with an imaginary node endowed with the average properties of all the nodes described above.

The set of Equation (8) or (9) with N=50 was solved using an explicit 4th-order Runge–Kutta scheme [13], properly adding the network term. We used this number of nodes because the Goodwin model is a macroeconomic model and, in the real world, there are not thousands of macroeconomies interacting between them, so a small network is closer to the types of economies that we are trying to characterize.

## 3. Results and Discussion

The mathematical model and the main parameters were introduced in the previous section. In particular, the intrinsic dynamics for each economy are controlled by the values of $\beta_{u}$ and $\beta_{v}$. On the other hand, we have several parameters controlling the type of interaction between the different economies. g controls the weight of the network on each economy and the type of network considered (BA, WS, random, or mean field). These are the parameters that we analyze below.

### 3.1. Phase Diagram for a Complex Network Model

**Figure 4:**
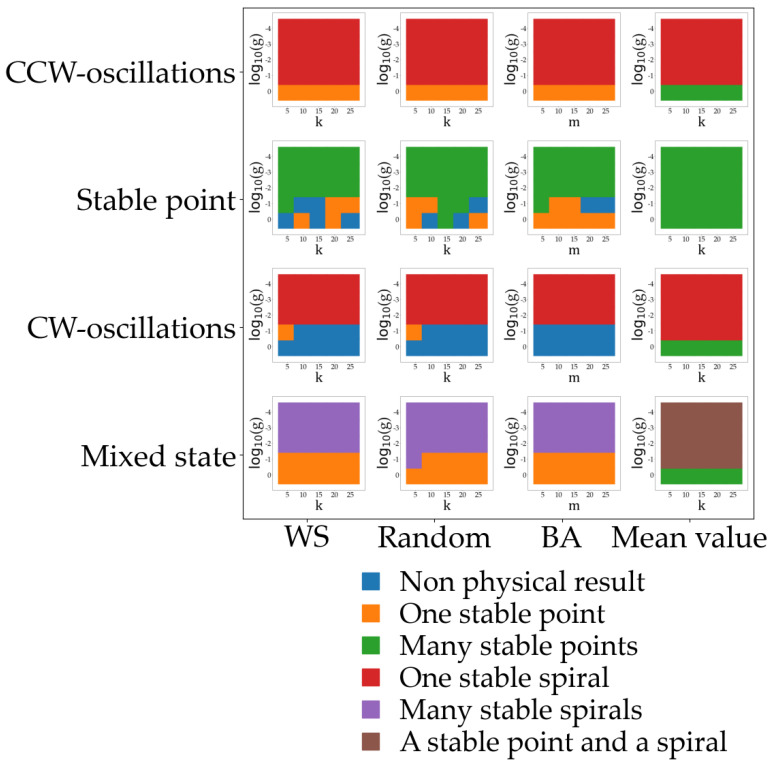
Multiple phase diagrams for the relevant variables in our model. Color-coded are marked the final configurations after the transient for the different values of the relevant parameters considered. (All other parameters are set equal to 1 as in previous results). Note that for each point in the phase diagrams multiple simulations were performed in order to assure the result as the specific network considered depends on the simulation (between 5 and 10 simulations were typically run).

Figure 4 summarizes all the behaviors observed for this model. Each panel in the figure corresponds to a phase diagram where the weight of the network term g and the connectivity k of the network are varied (with the exception of the BA, where the parameter is m, which is the number of connections that a node must have when the network is being created out of a random network; see the Supplementary Materials for details). Sixteen of such diagrams are presented, each corresponding to a different type of network (WS, random, BA, and mean field) as we move along the horizontal direction, whereas, when moving along the vertical direction, we explore different values of the parameters $\beta_{u}$ and $\beta_{v}$ that control the internal dynamic of each economy.

For these parameters, we considered the three dynamical behaviors described in Figure 2 and Figure 3 that present non-divergent behaviors, namely CW-oscillations or clockwise oscillations ($\beta_{u}=0.5$ and $\beta_{v}=0.5$), where all the economies exhibit this behavior in the absence of the network influence; stable point ($\beta_{u}=1.5$ and $\beta_{v}=0.5$), where all economies are in a fixed steady point in the absence of a network; CCW-oscillations or counterclockwise oscillations ($\beta_{u}=1.5$ and $\beta_{v}=1.5$), where all economies oscillate in the absence of a network. The last configuration considered and named as mixed state in Figure 4 corresponds to a situation where each individual economy has a different value of $\beta_{u}$ and $\beta_{v}$ randomly chosen from the three configurations described above.

The collective behaviors observed are color-coded in Figure 4. Regions in blue denote those parameter values that result in non-physical configurations (divergent trajectories mostly). The rest of the collective behaviors are classified depending on the collective behavior of the system variables. For some parameter values (marked in orange), we observe that all variables converge to a common fixed steady state. Under some other circumstances, each economy or node in our network converges with time to a different steady state (marked in green). Two other states are observed involving some transient oscillations in the process of reaching a stationary state. In red, we mark those parameter values that produce a solution where all the economies oscillatory synchronize and collectively tend to a single fixed point (spiral stable state). In addition, those parameter values that induce several spiral states, different for each node, are marked in purple. Finally, marked in brown are those parameter values that result in a complicated behavior where each node follows the dynamics of a fixed point or a stable spiral. For those cases where we obtained a non-physical solution, we repeated the simulations to discard numerical instabilities.

Note that the effect of the network connectivity is almost negligible for all the cases considered. The network weight, given by parameter g, on the other hand, determines the dynamic of the solution.

Four different network topologies were considered, and the results are independent of them. Only the mean field case (described by (Equation (9)) exhibits a different behavior. For the three other networks (described by Equation (8)), the salary always goes to the fixed point predicted by the linear approximation; on the other hand, and for the main field case (Equation (9)), we observe multiple fixed points different for each node. As we will discuss later, the fixed point reached by the economies can be related to the specific connectivity degree of each node.

The small number of economies considered (N=50) might be the reason behind this lack of sensitivity toward the selected network. A larger number of involved economies are expected to be more sensitive to the network configuration although, for the present study, we stuck to smaller numbers that better describe the interaction of a limited number of macroeconomies.

The internal dynamic of each economy turned out to be determinant in the selection of the final configuration. It is interesting to note that economies endowed with a counterclockwise initial condition evolve into a steady state due to the network influence.

**Figure 5:**
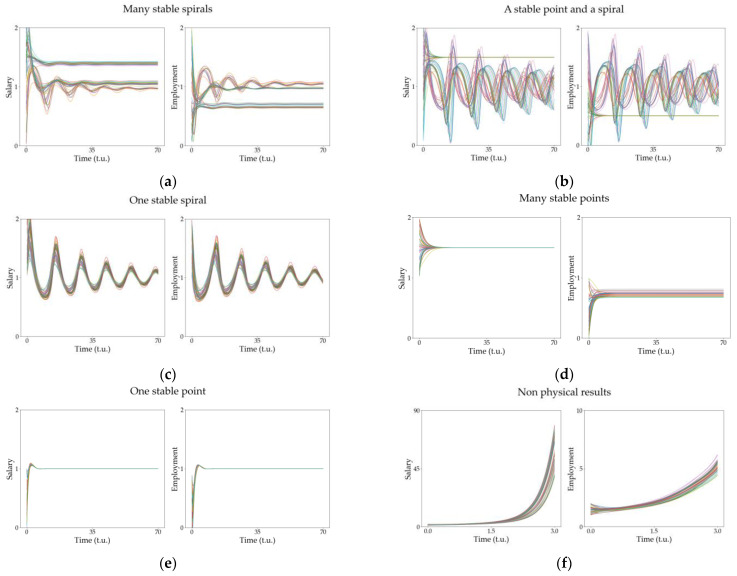
Evolution of the model variables for the different configurations described in Figure 4. The temporal evolution of the two model variables is plotted when the initial configuration of the nodes is (**a**) many stable spirals, (**b**) a stable point and a spiral, (**c**) one stable spiral, (**d**) many stable points, (**e**) one stable point and (**f**) corresponds with a non-physical result. Note that, in (**f**), the scale in the vertical axis is significantly larger than in the rest of the figures.

In Figure 5, we present an example for each of the dynamical behaviors represented in Figure 4. Going from left to right and up to down in Figure 5, the first row presents the dynamics observed for the two model variables when a mixed state is considered as the initial condition, many stable spirals (Figure 5a), and a stable point and a spiral (Figure 5b). In this last case, the fixed point is always $\left(u=1.5,v=0.5\right)$, and the stable spiral node is $\left(u=1,v=1\right)$. In both cases, the final state is a fixed point that is reached via some temporal non-coherent damped oscillations. Note that even those economies that were initially already in a steady state experience some oscillations before reaching the stability.

Figure 5c shows the dynamic of the system when all economies converge to a fixed point after several damped oscillations when the initial state is CW-oscillations. Note that the cycles (or centers) of the Goodwin model disappear due to the network influence stabilizing a fixed point. In Figure 5d, all economies steadily move to a fixed point that changes depending on the particular node (this case will be analyzed in more detail below).

In Figure 5e, all economies exhibit a single fixed point after some transient that does not involve damped oscillations. Note that this configuration is observed for large values of g, i.e., when the weight of the connected economies is important. From an economic point of view, all the economies are so interconnected that the final solution is common to all of them; they describe a common global economy. Finally, Figure 5f is an example of a non-physical solution where all economies diverge to infinity due to the high coupling with the network. This non-physical solution is observed when the parameter quantifying the weight of the network (g) is significantly large.

Note that the system becomes more sensitive to the initial conditions for each economy and the network topology when the network coupling is very strong (cases where $\log_{10}g\leq -1$). When the economies are less coupled, the structure of the network becomes less relevant. In addition, the oscillatory behavior observed in our solutions is the natural approach for reaching the stationary solution in our model and it seems a plausible solution in a more realistic context. However, it is interesting to note that the direct relaxation to the stationary solution is also a solution in the model (Figure 5d).

In the following sections, we analyze the main collective phenomena observed in the network of connected economies, specifically the synchronized oscillations of the solutions in Figure 5c and the relation of the fixed points in employment with the connectivity in the solutions of Figure 5d.

### 3.2. Synchronization of the Spiral Solutions

**Figure 6:**
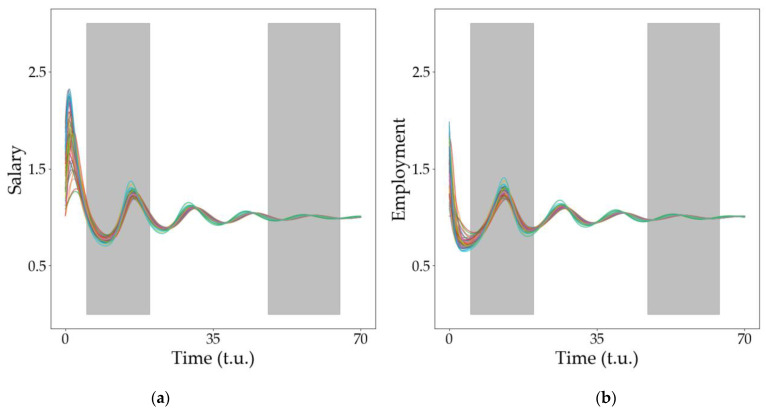

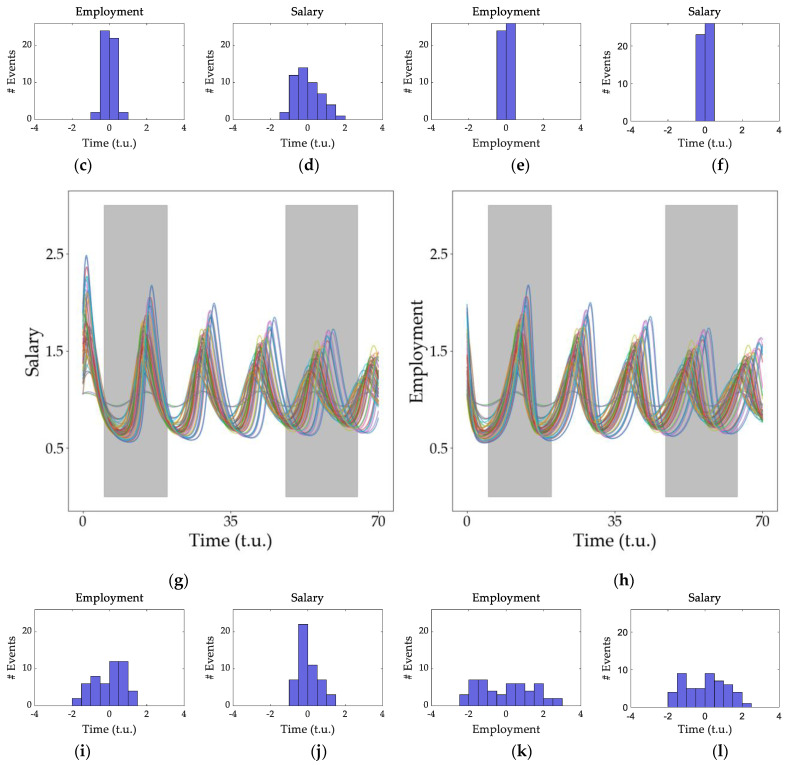
Synchronization in spiral dynamics. (**a**,**b**) Evolution of our economic network variables for a large network weight ($\log_{10}g=-2$). The shaded areas are the regions where we will analyze the local maxima and study the difference in phases between economies. The histograms with the time delay between all economies are shown in (**c**) for the u variable in the first shadowed region and (**d**) for the v variable in the first shadowed region. The histograms at the end of the simulation are shown in (**e**) for the u variable and (**f**) for the v variable. Evolutions of (**g**) the employment variable (u) and (**h**) the salary variable (v) are shown for a low value of the network weight ($\log_{10}g=-3$). The behavior of all the economies is less synchronized as shown in the histograms in the lowest row. The histograms with the time delays between the economies whose dynamics are plotted in figures (**g**,**h**) are shown in (**i**) for the u variable in the first shadowed region and (**j**) for the v variable in the first shadowed region. The histograms at the end of the simulation are shown in (**k**) for the u variable and (**l**) for the v variable. The network used for this simulation is a WS with k=15 and p=0.05.

Figure 4 and Figure 5 show that, for some parameter values, all economies may lead to a fixed value but, during the transient, they may experience synchronized damped oscillations. We analyze this behavior in this section. Figure 6 shows a summary of the results observed. Figure 6a shows the evolution of the variable u (salary) for all the economies considered and the evolution of the variable v (employment) is shown in Figure 6b. Note that, although the initial values for the simulations are randomly chosen, all the economies synchronize almost immediately and coherently oscillate. The lower row in Figure 6 (Figure 6c–f) shows the histograms of the delays between the different economies and model variables shortly after the beginning of the simulation and at some later stage. Note that, in both instances, the histogram has a narrow bell-shaped distribution, although the distribution becomes narrower as time passes and the different economies interact for longer periods (Figure 6e,f). Figure 6g,h show the evolution of variables u and v when the network weight is one order of magnitude smaller. In this case, the synchronization is not evident, and the histograms presented in the following row (Figure 6i–l) show a much broader distribution (that becomes even broader as time evolves), reflecting the non-coherent state of synchronization between the economies considered.

**Figure 7:**
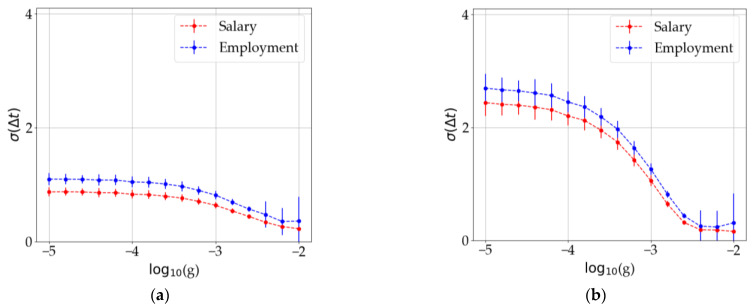
Dispersion of the economies’ variables as a function of the network weight g. (**a**) Standard deviation at the beginning of each simulation. (**b**) Same magnitude calculated at the end of the simulation. Error bars show the dispersion of this parameter over 100 runs of the same simulation.

A narrow distribution reflects that the economies oscillate in synchrony whereas a wider-spread histogram corresponds to a set of unsynchronized economies. An interesting parameter for characterizing this is the standard deviation from the mean value, σ. We analyzed the variation in σ (that, in our context, gives an inverse measurement of the synchronization degree) as we varied the weight of the network on the dynamics, g. These results are shown in Figure 7. Note that, as expected, as the weight of the network becomes more important, the degree of synchronization among the economies becomes higher (and thus σ becomes smaller). Figure 7a shows that the variation in g produces unsynchronous oscillations.

Our results also show some dependence on the average degree of the connectivity for each type of network considered, but the results are not conclusive. Nevertheless, as they might help to illustrate the global behavior, we include them in the Supplementary Information.

Different network topologies produce similar results to those in Figure 7 (not shown in the text).

### 3.3. Dispersion of the Steady States

**Figure 8:**
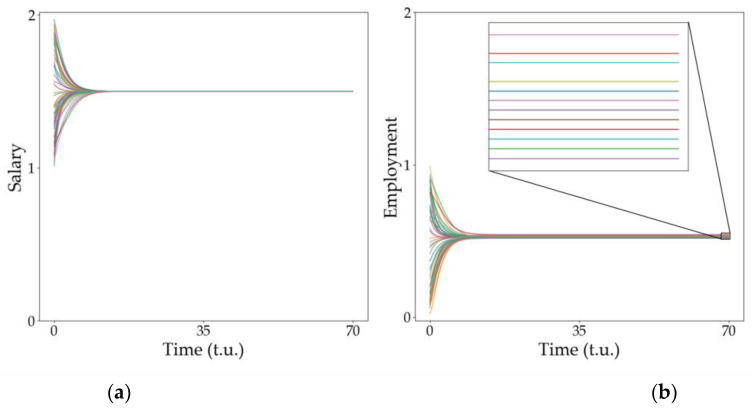

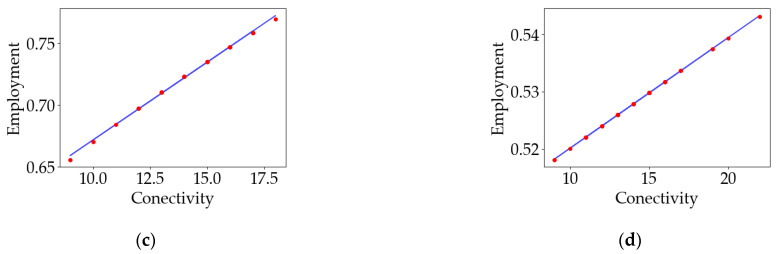
Relationship between connectivity and the stable point. Evolution of (**a**) variable u, salary, and (**b**) variable v, employment, for all the economies in the network. In (**b**), there is a zoom on the squared section to better appreciate the distance between the stable points. Dependence of the employment final state with the node connectivity for (**c**) a large value of the network weight ($\log_{10}g=-2$) and for (**d**) a smaller value of the network weight ($\log_{10}g=-3$). All simulations used a random network with k=15. The size of the vertical axis is different for each figure to improve visibility of the results.

Another interesting phenomenon observed in Figure 4 and Figure 5 (specifically, in Figure 5d) is observed when the economies tend toward a steady value but each economy (node in the network) reaches a significantly different steady state. This is observed in Figure 8. In Figure 8a, we can notice that, as the variable salary reaches the same value in all nodes, the other variable, employment, differs depending on the node (Figure 8b). In order to enlighten the origin of this deviation, we measured the connectivity of each node (economy) and plotted the corresponding value of the employment variable at the steady state. The results are plotted in Figure 8b,c. There is a clear linear dependence between both variables and thus we conclude that the final value of the variables linearly depends on the node connectivity. This means that those economies more connected to others will benefit from larger levels of employment while the salaries will remain unchanged but equal to all economies in the network.

As in the previous section, when we reduce the value of g, the interaction with the network becomes weaker, which implies that the steady states merge together and the distance between them becomes negligible. Note that the results presented in this section correspond to a random network, but equivalent results are observed with the other types of networks considered (see the results in the Supplementary Materials). This lack of sensitivity to the network structure was already expected from Figure 4, where the effect of the type of network on the type of behavior observed is demonstrated to be almost null. Note that, from an economic point of view, our results indicate that increasing the connectivity of each node (economy) results in a rise in the employment variable as well, meaning that very global economies are more prone to rising salaries in all economies involved.

## 4. Conclusions

The modified Goodwin model, which introduces some limiting values for the salary and employment variables, results in an adequate mathematical description for a simplified economy and long-time observations. In particular, it prevents divergent solutions. The analysis of a reduced number of different economies coupled via some network of connections showed interesting collective properties. The different parameters relevant for this study were analyzed and the importance of the neighboring economies was stressed. The specific topology of the network considered appears to be less relevant than expected, probably due to the small number of economies considered. Nevertheless, the connectivity of the nodes plays an important role in determining the levels of employment and salary achieved. The strength of the network coupling also proves to be determinant in controlling the dynamics. Two phenomena are described in more detail: the synchronous oscillatory behavior that strongly depends on the strength of the interactions between the nodes of the network and the dispersion of the final steady states that is determined by the connectivity degree of each node. The economic descriptors of each economy are strongly influenced by the other economies connected. It is interesting to note the case of strongly coupled economies that result in a global improvement of the model variables.

It is important to note that, although the model describing each economy is a simple one that lacks the complexity of the real economies, the aim of this contribution focuses on the synergetic behavior of a collection of economies interconnected. In fact, the existence of a network topology reflects the fact that we are not in a global fully interconnected economy and allows us to examine the role of the different parts of the structure on the variables describing each economy. Within the structure described here, it is possible to imagine modifications of the present model for a more realistic description of the reality. In any case, the basic consequences derived from our contribution remain valid, i.e., the network structure formed by the different economies strongly influence the dynamic of the whole economic system.

In summary, we can conclude that the interactions between economies, rather than being negligible, may become the source of the dynamic for the entire system, and that the structure of the network also plays a significant role in the collective behavior.

## Supplementary Materials

The following supporting information can be downloaded at: https://www.mdpi.com/article/10.3390/e25060894/s1.
