# Supplementary material for: A Goodwin Model Modification and Its Interactions in Complex Networks
Source: Entropy (Basel). 2023 Jun 2;25(6):894. doi: 10.3390/e25060894 (PMC10297564; doi:10.3390/e25060894)
Supplement: Supplementary file 1 [file entropy-25-00894-s001.zip › entropy-2336910-supplementary.pdf]

Supplementary Material

A Goodwin model modification and its interactions in complex networks

Francisco Yáñez-Rodríguez [1] and Alberto P. Muñuzuri [1,2,*]

[1] Group of NonLinear Physics, Univ. of Santiago de Compostela, 15706 Santiago de Compostela (Spain)

[2] Galician Center for Mathematical Research and Technology (CITMAga), 15782 Santiago de Compostela, Spain

[*] Author to whom correspondence should be addressed: alberto.perez.munuzuri@usc.es

S1.- Original Goodwin model: simulations, linear stability analysis and phase diagrams

In the following, we present the derivation of the equations of the Goodwin model.

In equation (1) the nonlinear differential equations that Goodwin assumed as true to derive the rest of the model equations are presented. The first is the so-called Philips curve [19], and the second is an assumption of the model itself, similar to the previous one,

$$\frac{dw}{dt} = w(\rho v - \gamma)\,;$$
$$\frac{dc}{dt} = q(1-u) = c\left(\frac{1-u}{\kappa}\right) \qquad (1)$$

As we see in equation (1), the structure of both equations is similar. An increase in both variables produces a fast raise or decrease, depending on the sign of the parenthesis. For $v > \gamma/\rho$, w will increase, and vice versa in the opposite situation. The contrary is observed for c: for $u > 1$, its value will diminish.

Also, as we mentioned in the main text, Goodwin defined the increase of productivity and population as an exponential growth, so their differential equations are $p' = \alpha\, p$ and also $n' = \delta\, n$ with $p' = \frac{dp}{dt}$ in all equations here considered.

With these two assumptions, we derive the equations for the rest of the variables of the model, which we will develop in the following equation (2),

$$l = \frac{q}{p} \rightarrow l' = \frac{q'p - p'q}{p^2} = \frac{p}{p^2}\left[q\left(\frac{1-u}{\kappa}\right) - \alpha q\right] = \frac{q}{p}\left(\frac{1-u}{\kappa} - \alpha\right); l' = l\left(\frac{1-u}{\kappa} - \alpha\right) \qquad (2)$$

$$v = \frac{l}{n} \rightarrow v' = \frac{l'n - n'l}{n^2} = \frac{n}{n^2}\left[l\left(\frac{1-u}{\kappa} - \alpha\right) - \delta l\right]; \boxed{v' = v\left(\frac{1-u}{\kappa} - \alpha - \delta\right)}$$

$$u = \frac{w}{p} \rightarrow u' = \frac{w'p - p'w}{p^2} = \frac{p}{p^2}[w(\rho v - \gamma) - \alpha w] = \frac{w}{p}(\rho v - \gamma - \alpha); \boxed{u' = u(\rho v - \gamma - \alpha)}$$

Now, with the final expressions for u' and v', we will calculate the behavior of these equations using the lineal approximation,

$$v' = u' = 0 \rightarrow \vec{x}_1 = (u = 0, v = 0)\; ;\; \vec{x}_2 = \left(u = \frac{1 - \kappa(\alpha + \delta)}{\lambda}, v = \frac{\gamma + \alpha}{\rho}\right)$$

$$\mathcal{J}(u, v) = \begin{pmatrix} \partial_u u' & \partial_v u' \\ \partial_u v' & \partial_v v' \end{pmatrix} = \begin{pmatrix} \rho v - \gamma - \alpha & u\rho \\ -\frac{\lambda v}{\kappa} & \frac{1 - \lambda u}{\kappa} - \alpha - \delta \end{pmatrix} \tag{3}$$

$$|\mathcal{J}(\vec{x}_1) - \mu\mathbb{I}| = \begin{pmatrix} -\gamma - \alpha - \mu & 0 \\ 0 & \frac{1}{\kappa} - \alpha - \delta - \mu \end{pmatrix}; \begin{matrix} \mu_1 = -\gamma - \alpha \\ \mu_2 = \frac{1}{\kappa} - \alpha - \delta \end{matrix}$$

$$|\mathcal{J}(\vec{x}_2) - \mu\mathbb{I}| = \begin{pmatrix} -\mu & \rho\left[\frac{1 - \kappa(\alpha + \delta)}{\lambda}\right] \\ \frac{\lambda}{\kappa}\left(\frac{\gamma + \alpha}{\rho}\right) & -\mu \end{pmatrix}; \mu_1, \mu_2 = \pm i\sqrt{\left(\frac{1}{\kappa} - \alpha - \delta\right)(\gamma + \alpha)}$$

We can see how the model will have a bifurcation when $\kappa^{-1} = \alpha + \delta$ (remember that all the parameters are defined strictly positive). Looking at the sign of these eigenvalues, we see that we have one regime where there are periodic solutions and a saddle point ($\kappa^{-1} > \alpha + \delta$), and other regime where there is a saddle point and a stable point at the origin. We will represent these two configurations in Figure S1.

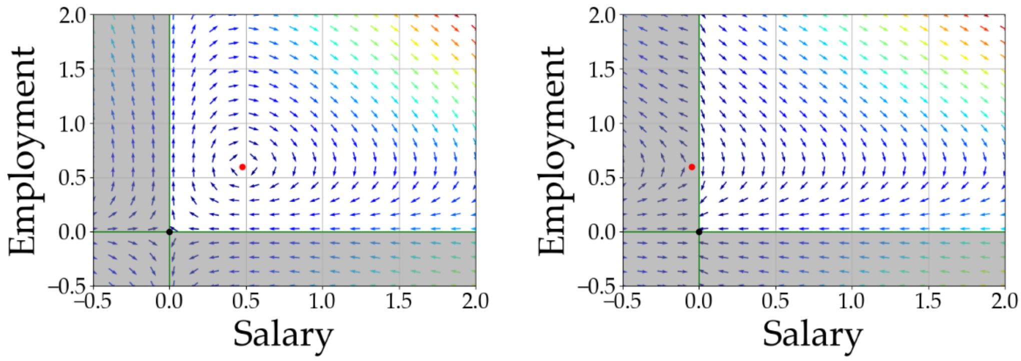

Figure S1. Flow diagram that shows the dynamics of the two behaviors observed in the original Goodwin model, analogue to Figure 2 in the main paper. Notice that only the first diagram is considered a solution with economic meaning. The second one is just a mathematical solution without physical meaning.

We observe in Figure S1 the known periodic solutions for the Goodwin model. Note that the fixed-point solutions (with a stable point at the origin) are a trivial solution as they correspond with a situation without employment and, thus, salary.

If we graph the phase diagram for κ and the sum of $\alpha + \gamma$, we obtain the figure S2: The red region is the region with a stable point solution, while the blue region maks the set of parameter values for the periodic dynamic.

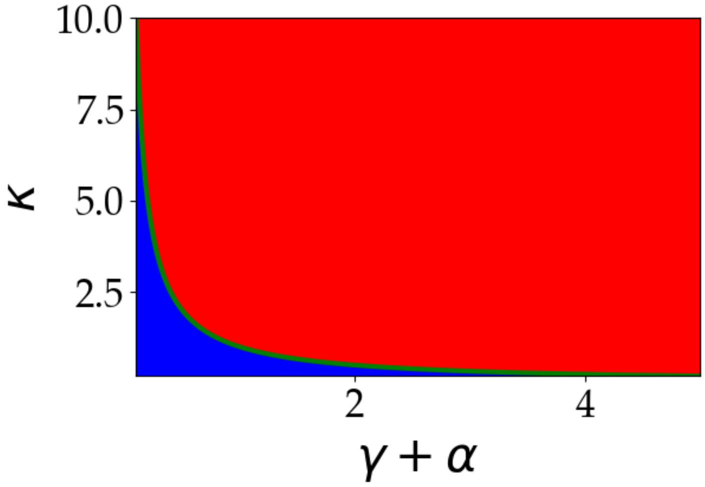

(a)

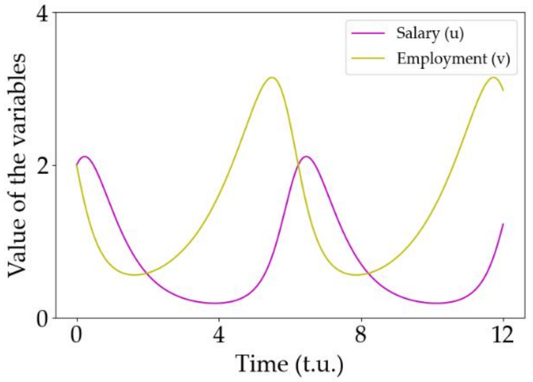

(b)

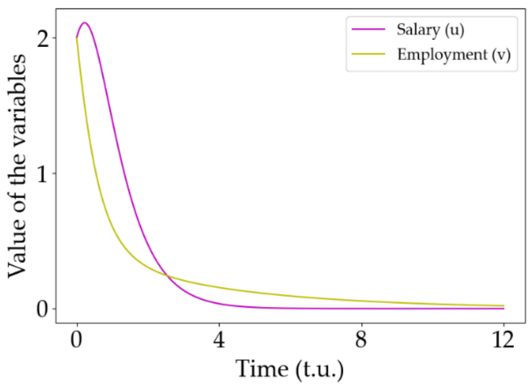

(c)

Figure S2. (a). Phase diagram that shows the different regimes present in the model. The red region is the set of parameters where we obtain a stable point, and the blue region is a region where we obtain the characteristic periodic behaviors of the model. The green line that separates both regions represents a zone where we obtain a degenerate node, also without economic interest. (**b**). Evolution of the variables for an economy with parameters in the blue zone. In this case, the initial conditions are $u = v = 2$, $\gamma = 0.5, \alpha = 0.2$ and $\kappa = 1$. (c). Evolution of the variables for an economy with parameters in the blue region. In this case, the initial conditions are $u = v = 2$, $\gamma = 0.5, \alpha = 1.2$ and $\kappa = 1$

S2.- Examples of the networks used

In the main text, we had used different types of networks. Here we provide details of the networks used and explain the differences between them.

The first type of network is the Watts-Strogatz network ($WS(N,k,p)$). It is a network where all the nodes involved have connections with their closest neighbors except for some connections with distant nodes. It depends on three parameters: the number of elements in the network, N; the mean connectivity of the nodes, k; and the parameter p. The parameter p is a probability that a node has connections with a distant node. I.e. networks with $p\sim 0$ will be networks where nodes are only connected with the closest neighbors, and for those networks with $p\sim 1$, all nodes will be randomly connected with the others. In fact, along the manuscript, the random networks used will be WS with $p = 1$ in order to assure that the connectivity was kept constant. Figure S3a shows an example of a WS network and in Figure S3b the distribution of connectivities for this particular network. Figures S3c and S3d are an example of a WS network with $p =$ 1.

We also used a Barabási-Albert network $BA(N,m)$. This type of network has the property that the connectivity of their nodes has an exponential behavior; there are many nodes with few connections and vice versa, few nodes with many connections. Unlike the Watts-Strogatz network, the distance between nodes is not relevant in this kind of network. The unique factor that matters in the Barabási-Albert network (besides the number of nodes N) is the parameter m, which is the minimum of connections that a node can stablish. With low m, the exponential form of the connectivity is more remarkable. However, for highs values of m, the exponential form is more difficult to appreciate. Figure S3e is a graph of an example of the BA networks used along the main manuscript. Figure S3f is the connectivity's histogram for this network.

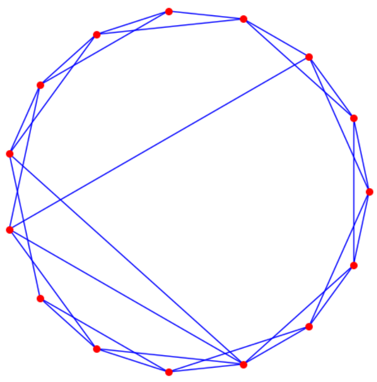

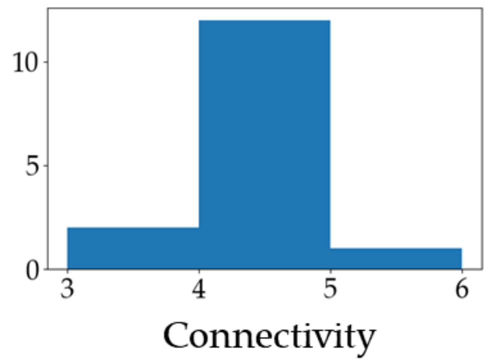

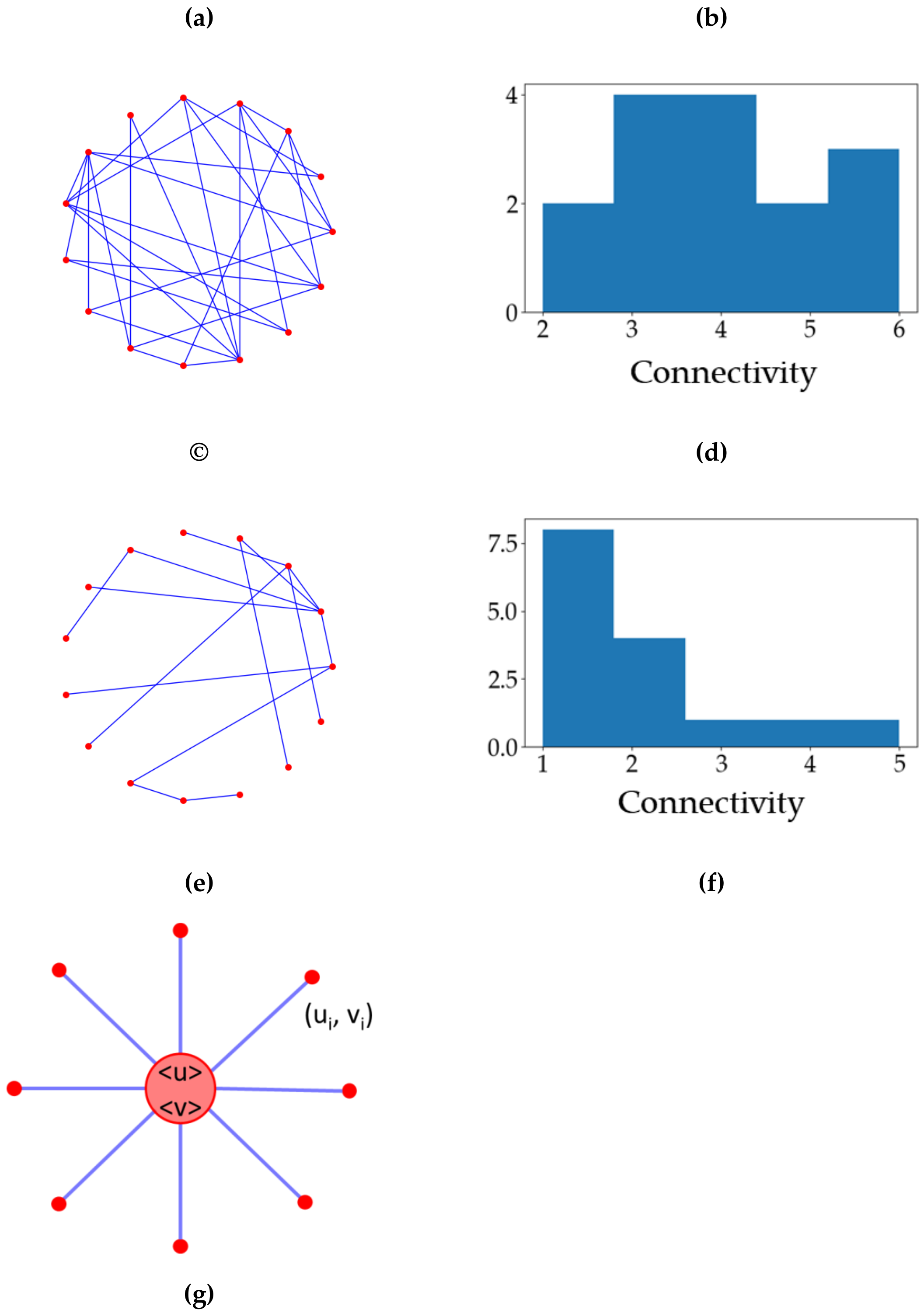

Figure S3. We represented a $WS(N = 15, k = 4, p = 0.05)$ in (**a**) and his connectivity histogram in (**b**). We did the same for a random network, that we computed as a $WS(N = 15, k = 4, p = 1)$; we represented it in (**c**) and his histogram connectivity in (**d**). Finally, we represented a $BA(N = 15, m = 1)$ in (**e**) and its histogram connectivity in (**f**).(g) Scheme of a mean field configuration. Note that only for these figures and for illustration purposes $N = 15$, for the calculations shown in the main text $N = 50$.

The final configuration used is a mean-field network that describes a situation where all the nodes in the network are connected to an imaginary node endowed with a value

for the variables equal to the average of the variable values for all the nodes. A scheme of this configuration is plotted in Figure S3g.

S3.- Connectivity in the spiral solutions

As shown in Figure 4 in the main paper, we obtain a spiral solution for values of $g \leq 10^{-2}$. Figure S4 shows the evolution of the Salary (Figure S4a) and Employment (Figure S4b) for a WS network of economies and for a set of parameters that warrants a spiral solution. Two areas are marked in gray at the beginning of the numerical simulation and at the end. We measure the maxima of each variable in both areas and plot them versus the time when the maxima are achieved. We color code each node depending on the connectivity of the node. The results are in Figure S4c to S4f. Note in Figure S4c and S4d that there is no correlation between the amplitude of the oscillation and the connectivity of that node. Nevertheless, as time evolves the results change as plotted in Figures S4e and S4f. Here, the moment in time when the maximum is reached correlates with the connectivity of the nodes, those nodes with less connectivity oscillating first.

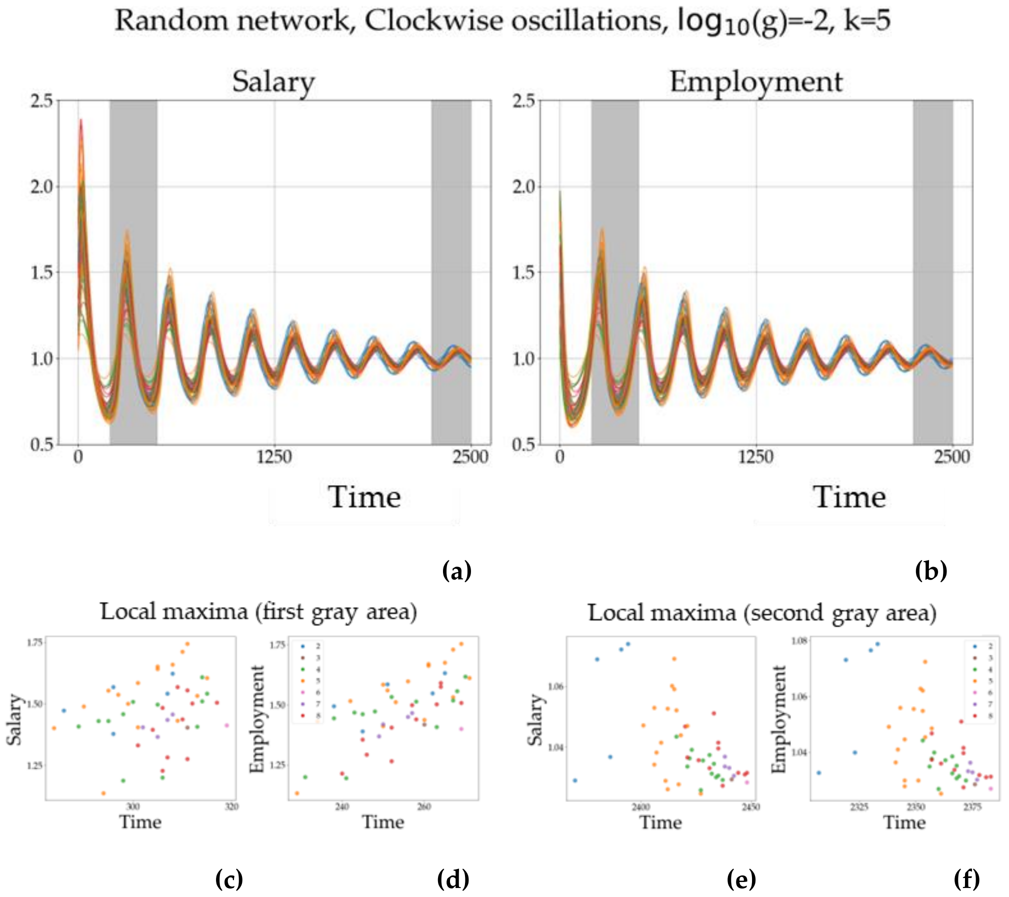

Figure S4. Synchronization of the spiral solutions. Time evolution of **(a)** the Salary variable for all nodes (economies) in the network and of **(b)** the Employment variable. Areas in gray mark the regions where maxima are evaluated. **(c)** and **(d)** plot Salary and Employment maxima values and the time they happened at the beginning of the simulation (first gray area). The

color of each dot reflects the connectivity of that particular node. **(e)** and **(f)** shows Salary and Employment maxima and the time they happened at the end of the simulation (second gray area).

Increasing the average connectivity of the network, we further explore this phenomenon. The results are plotted in Figure S5 for both variables. At a final stage of the simulation, we plot the amplitude of the oscillations for each node and each variable (Salary in Figure S5a and Employment in Figure S5b). We observe that increasing connectivity of the node produces oscillations with larger amplitude that reach the maximum at a later moment. These results were confirmed using different types of networks (i.e. BA, not shown). Although the values of the oscillation's amplitudes measured are quite small, the tendency seems quite clear.

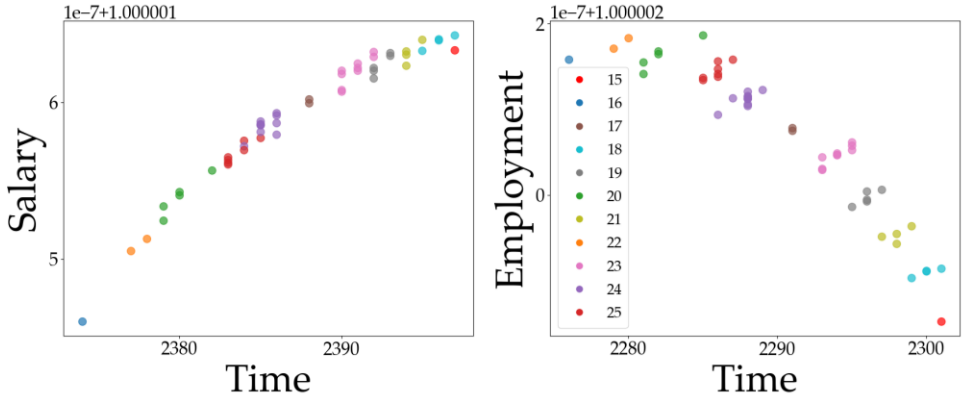

Figure S5. Maximum values for the **(a)** Salary and **(b)** Employment and the times when they happened measured at the end of a simulation The color for each do in the figure encodes the value of the connectivity for that specific node.

Decreasing the network weight (g) makes this synchronization harder to see as its origin is on the interaction with the network. In fact, for values of $g \approx 10^{-4}$, the synchronization becomes negligible. These observations are independent of whether the oscillations are clockwise or counterclockwise.

S4.- Dispersion of the economies' variables as a function of the network weight g for other types of networks.

In this section we show the analogue of Figure 7 in the main text for other types of networks.

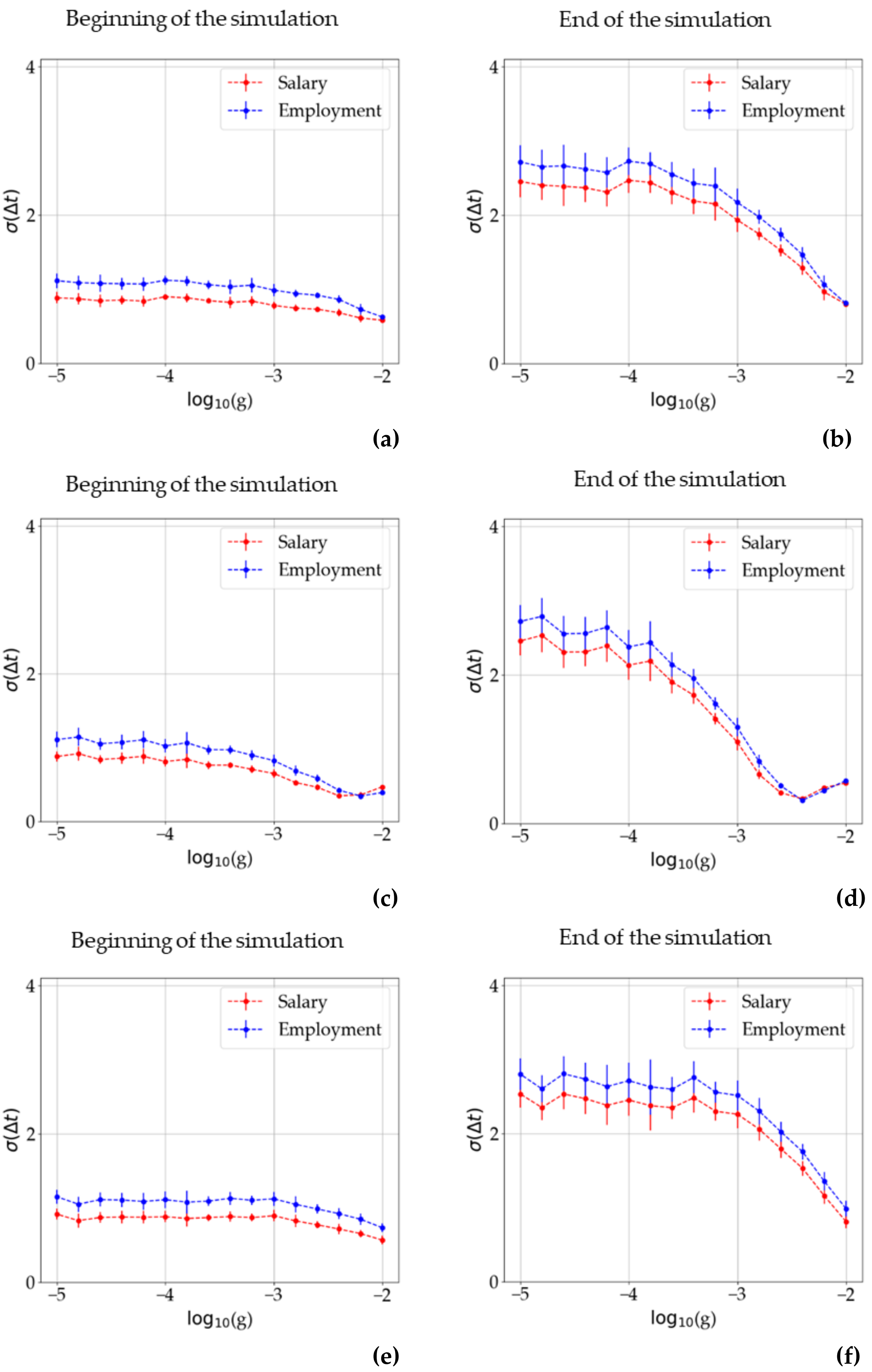

Figure S6. Dispersion of the economies' variables as a function of the network weight g for other types of networks (analogue of Figure 7 in the main manuscript). **(a)** and **(b)** consider a BA network ($N = 50, m = 2$). **(c)** and **(d)** use a random network ($N = 50,\ k = 15,\ p = 1$). **(e)** and **(f)** use a WS network ($N = 50,\ k = 2,\ p = 0.05$)

S5.- Linear dependence of the stable points in employment for other types of networks.

In the main text, we establish a linear dependence between the connectivity of each node in the network with the steady state reached. Those results were calculated using a WS network. In Figure S7, we plot equivalent results calculated using different network topologies.

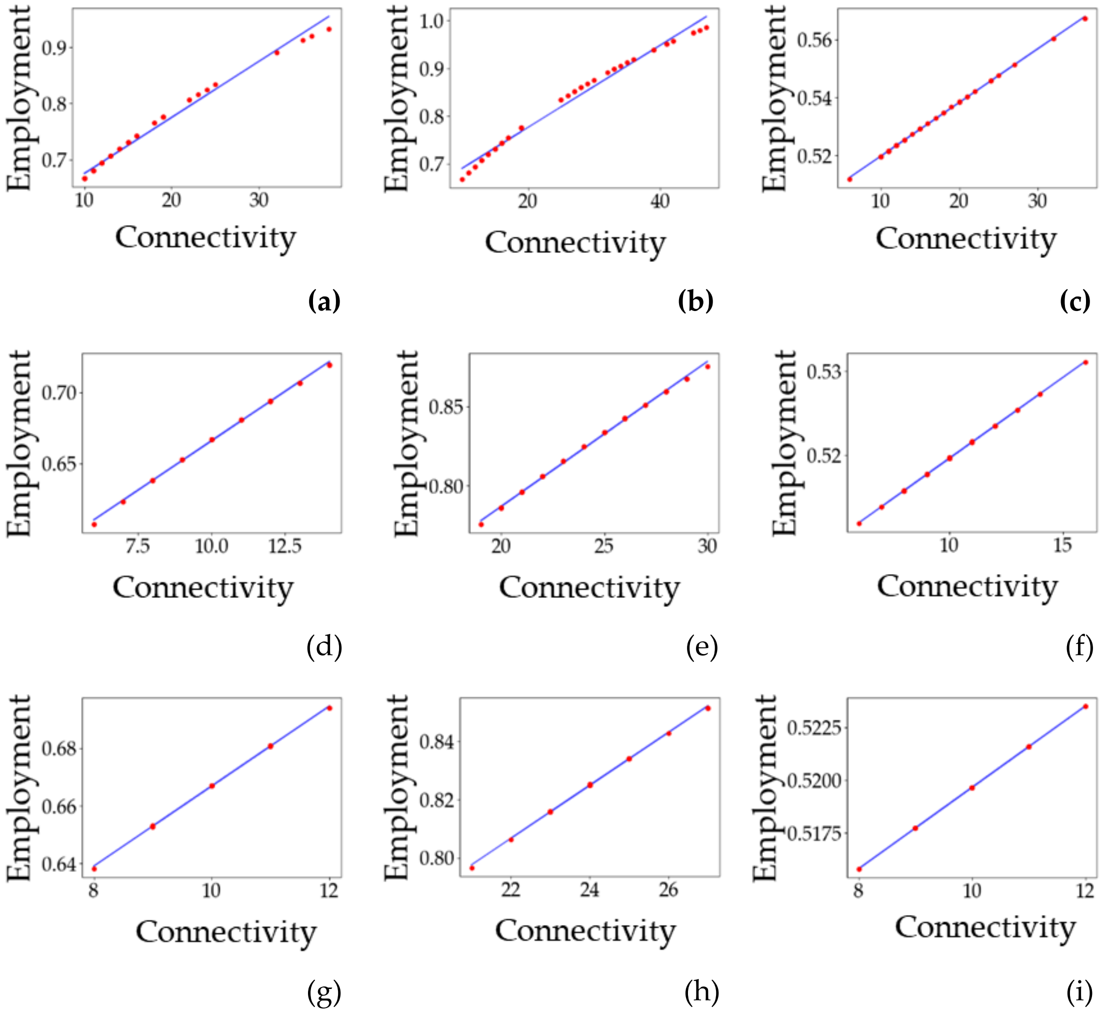

Figure S7. Linear dependence of the stable points in employment for other types of networks. **(a)** BA network with $\log_{10} g = -2$ and $m = 10$. **(b)** BA network with $\log_{10} g = -2$ and $m = 25$. **(c)** BA network with $\log_{10} g = -3$ and $m = 10$. **(d)** WS network with $p = 1$ (random network), $\log_{10} g = -2$ and $k = 10$. **(e)** WS network with $p = 1$ (random network), $\log_{10} g = -2$ and $k = 25$. **(f)** WS network with $p = 1$ (random network), $\log_{10} g = -3$ and $k = 10$. **(g)** WS network with $p = 0.01$, $\log_{10} g = -2$ and $k = 10$. **(h)** WS network with $p = 0.01$, $\log_{10} g = -2$ and $k = 25$. **(i)** WS network with $p = 0.01$, $\log_{10} g = -3$ and $k = 10$.
